# Establishment of stable Vero cell lines expressing TMPRSS2 and MSPL: A useful tool for propagating porcine epidemic diarrhea virus in the absence of exogenous trypsin

**DOI:** 10.1080/21505594.2020.1770491

**Published:** 2020-05-29

**Authors:** Xiaona Wang, Xinyuan Qiao, Ling Sui, Haiyuan Zhao, Fengsai Li, Yan-Dong Tang, Wen Shi, Yuyao Guo, Yanping Jiang, Li Wang, Han Zhou, Lijie Tang, Yigang Xu, Yijing Li

**Affiliations:** a College of Veterinary Medicine, Northeast Agricultural University, Harbin, P.R. China; b Heilongjiang Key Laboratory for Animal Disease Control and Pharmaceutical Development, Harbin, P.R. China; c Department of Swine Breeding, Jiangsu Hanswine Food Co., Ltd, Ma’anshan, Anhui Province, China; d State Key Laboratory of Veterinary Biotechnology, Harbin Veterinary Research Institute, Chinese Academy of Agricultural Sciences, Harbin, China; e College of Animal Science and Technology, Northeast Agricultural University, Harbin, P.R. China

**Keywords:** Porcine epidemic diarrhea virus, TMPRSS2, MSPL, Vero cell lines, isolation and propagation

## Abstract

Porcine epidemic diarrhea virus (PEDV) is the causative agent of porcine epidemic diarrhea, causing substantial economic losses to the swine industry worldwide. However, the development of PEDV vaccine is hampered by its low propagation titer *in vitro*, due to difficulty in adapting to the cells and complex culture conditions, even in the presence of trypsin. Furthermore, the frequent variation, recombination, and evolution of PEDV resulted in reemergence and vaccination failure. In this study, we established the Vero/TMPRSS2 and Vero/MSPL cell lines, constitutively expressing type II transmembrane serine protease TMPRSS2 and MSPL, in order to increase the stability and titer of PEDV culture and isolation *in vitro*. Our study revealed that the Vero/TMPRSS2, especially Vero/MSPL cell lines, can effectively facilitate the titer and multicycle replication of cell-adapted PEDV in the absence of exogenous trypsin, by cleaving and activating PEDV S protein. Furthermore, our results also highlighted that Vero/TMPRSS2 and Vero/MSPL cells can significantly enhance the isolation of PEDV from the clinical tissue samples as well as promote viral infection and replication by cell-cell fusion. The successful construction of the Vero/TMPRSS2 and Vero/MSPL cell lines provides a useful approach for the isolation and propagation of PEDV, simplification of virus culture, and large-scale production of industrial vaccine, and the cell lines are also an important system to research PEDV S protein cleaved by host protease.

## Introduction

Porcine epidemic diarrhea virus (PEDV) mainly infects the neonatal suckling pigs with up to 100% mortality rate and causes severe villous atrophy, watery diarrhea, vomiting, and dehydration [1]; the infection was first described in pigs in the United Kingdoms in 1971 [2] and its emergence in China was reported in the 1980 s [3]. Notably, vaccination failure and high death rate as observed in the 2010 Chinese outbreak, with the reemergence of a more virulent PEDV [4], resulted in huge economic losses for the pig industry. Subsequently, PED rapidly spread to more than 30 states of the United States, causing its first outbreak in 2013 and significant damage to the US pork industry [5], following which further outbreaks were reported in Canada, Mexico, Korea, Germany, and Belgium [6–10].

PEDV, the causative agent of porcine epidemic diarrhea, is an enveloped RNA virus of the genus *Alphacoronavirus*, family *Coronaviridae* [11]. The spike protein of PEDV is an important determinant of PEDV virulence [12] and viral mutation [13,14]. Guo *et al*. discovered a new subgroup (GII-c) of PEDV by analyzing the complete genomes of 409 strains of the virus, from different countries, which upsurged in April 2010 and evolved from a recombination event between the GI-a and GII-a subgroups [15]. Based on the fact that the emergence and reemergence of PEDV eludes current vaccine strategies [16], exploring the mechanism of PEDV infection, establishing the effective isolation and culture methods, and developing new effective drugs and highly effective vaccine products may play a key role in preventing pigs from acquiring the PEDV infection. Nevertheless, low cell-propagation titer and difficulty in isolation and culture of PEDV epidemic strains are the major challenges to further the study of PEDV. Therefore, it is an unmet need to improve the propagation titer of the PEDV *in vitro*.

Generally, propagation of PEDV isolates *in vitro* requires exogenous trypsin addition, which facilitates cell-to-cell fusion [17], multicycle replication, and the release of PEDV virions clustered on the cells after replication [18]. Nonetheless, the propagation of PEDV *in vitro* still remains a major challenge owing to its low viral propagation titer, complex culture conditions, and gradually declining viral infectivity during the serial passages [19–21], thereby restricting the replication and culture of these virus *in vitro*. Additionally, exogenous trypsin damages the cells and the virus becomes subsequently insensitive to tryptase with further mutation and evolution [22], which has serious effects on the propagation titer of the virus and hinders the research of PEDV vaccine for disease prevention. Therefore, it would have great practical significance if a method is devised to effectively increase the propagation stability and the titer of PEDV cultured *in vitro*.

Recently, the type II transmembrane serine protease 2 (TMPRSS2) and MSPL, a trypsin-like serine protease, have gained increasing attention. TMPRSS2 has been found to facilitate the multicycle replication and spread of human influenza viruses in the absence of trypsin [23], activate the spike protein and subsequent induction of cell fusion in severe acute respiratory syndrome coronavirus (SARS-CoV) [24], and facilitate trypsin-independent porcine epidemic diarrhea virus replication in Vero cells [25]. Previous studies have reported that the constitutive expression of TMPRSS2 in the host cells could cleave the fusion protein of HMPV efficiently, further enhancing virus multiplication [26] and facilitating the release of viruses from the infected Vero cells [18], and thus support trypsin-independent influenza A viruses (FLUAVs) spread in the infected hosts or infected host cells [27]. Moreover, recent evidence suggests that MSPL can activate the spike proteins of MERS-CoV and SARS-CoV and can contribute to viral spread in the host [27]. Shulla *et al*. discovered that TMPRSS2 specifically facilitate SARS-CoV entry [28], and Heald-Sargent and Okumura et al. speculated that MSPL may be linked to the SARS-CoV entry due to cleaving influenza HAs [29,30]. Recent evidence suggested that TMPRSS2 and MSPL facilitated the entry of PEDV by cleaving the PEDV S protein [25]. However, the effects of TMPRSS2, especially MSPL, on the entry and replication of PEDV, animal coronaviruses, have not been thoroughly studied compared to other coronaviruses. Thus, we were curious to know whether TMPRSS2 and MSPL could replace trypsin for propagating PEDV *in vitro*.

In this study, in order to optimize the culture methods and enhance the propagation of PEDV *in vitro*, Vero cell lines stably expressing TMPRSS2 (Vero/TMPRSS2 cells) and MSPL (Vero/MSPL cells) were established, utilizing the third-generation lentivirus system (including FUGW, pMD2.G, and psPAX2 plasmids). The stable expression of TMPRSS2 and MSPL genes was confirmed by reverse transcription PCR, fluorescence observation, and western blot assay. The establishment of stable Vero/TMPRSS2 and Vero/MSPL cell lines was confirmed by FACS assay, in serially passaged cells. The effects on replication and multicycle growth of PEDV, including cell culture-adapted PEDV LJB/03 P23 (after 23 serial passages in cell cultures) and PEDV new isolate 2013-A and NJ in Vero/TMPRSS2 cells, Vero/MSPL cells, and Vero cells (with or without trypsin supplementation) were compared. Our results revealed that Vero/TMPRSS2 and Vero/MSPL cells effectively support PEDV propagation, multicycle growth by activating S protein and facilitate isolation of PEDV clinical samples *in vitro* without trypsin supplementation. The promoting effects of Vero/MSPL and Vero/TMPRSS2 cells on the replication of PEDV are substantially higher than those of exogenous trypsin. Of them, the effect of Vero/MSPL is more prominent, suggesting a promising approach for isolating and propagating PEDV *in vitro*.

## Materials and Methods

### Cells and viruses

The swine intestinal epithelial cell (IEC) line, established by Wang et al. [31], was kindly provided by Prof. Yanming Zhang (College of Veterinary Medicine, Northwest A&F University, Yangling, China), and was used to isolate and propagate PEDV [32,33]. The human embryonic kidney 293 T cells (HEK293 T, ATCC, Manassas, VA, USA), IEC and Vero cells (ATCC, Manassas, VA, USA) were cultured in Dulbecco’s modified Eagle’s medium (DMEM; Gibco, Grand Island, NY, USA) with 10% fetal bovine serum (Gibco). PEDV LJB/03 kept in our laboratory [34] was grown in Vero cells. In brief, PEDV LJB/03 was propagated (multiplicity of infection, MOI = 0.01) on the confluent cell monolayer for 1 h at 37°C under 5% CO_2_, supplemented with 3 µg/mL of trypsin (Gibco). After washing twice with sterile phosphate-buffered saline (PBS; pH 7.2), the cells were maintained in DMEM with 3 µg/mL trypsin. Cell cultures were collected until the cytopathic effect (CPE) exceeded 80%, and the supernatants were harvested by freeze-thaw method and stored at −80°C until required. PEDV isolates 2013-A and NJ that were isolated in our laboratory, by Heilongjiang in 2013 and Nanjing in 2011 [32], respectively. The small intestine tissues infected with PEDV were treated as follows. Briefly, intestinal tissue was frozen, ground with liquid nitrogen, and the ground tissue was suspended in DMEM solution and filtered using a 0.22 um filter. The separated tissues were stored at −80°C until further processing.

### Generation of Vero/TMPRSS2 and Vero/MSPL stable cell lines

**Figure 1.**
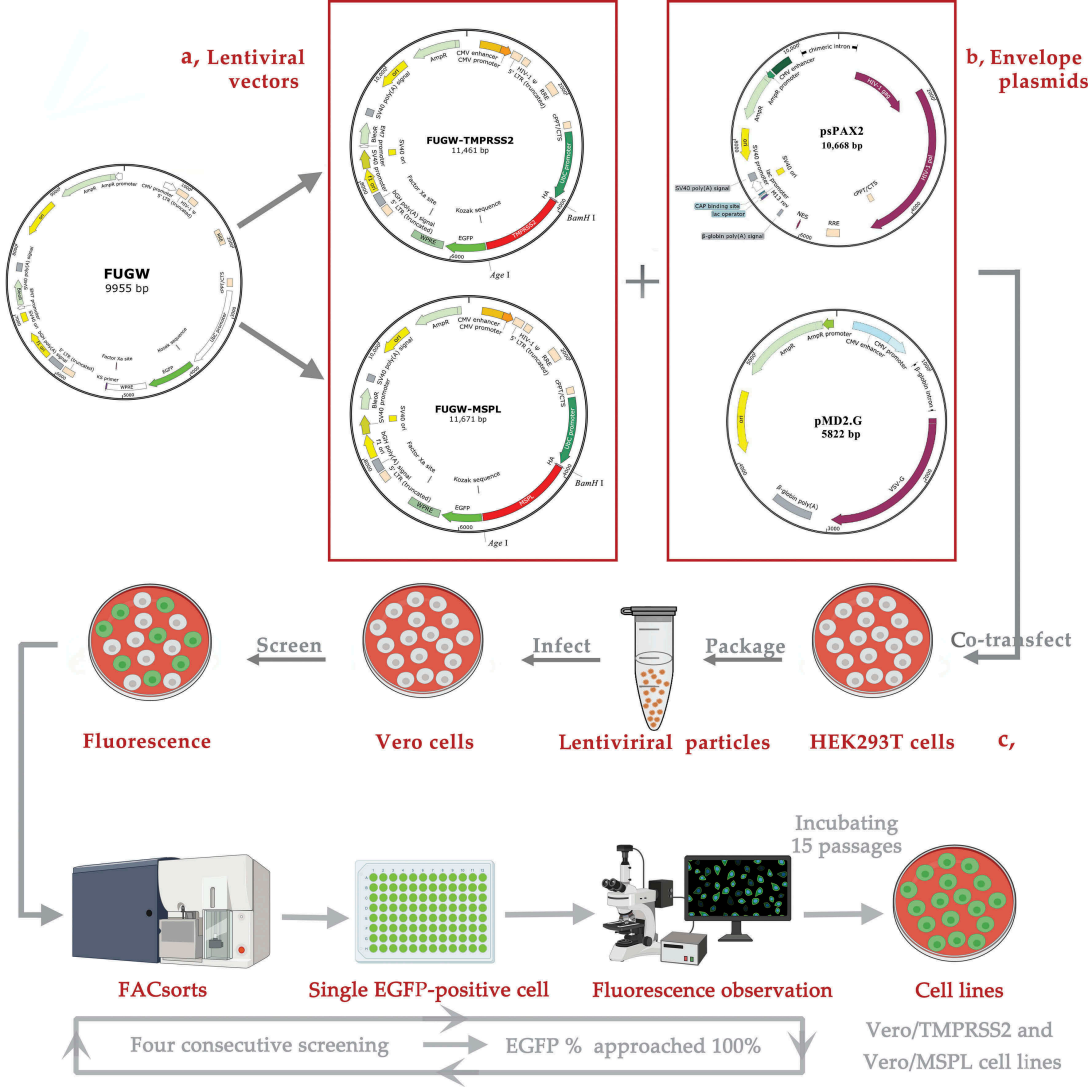
A schematic diagram for the construction of Vero/TMPRSS2 and Vero/MSPL cell lines, which were generated following the steps of arrows. (a) The constitutive recombinant plasmids (lentiviral vector) FUGW-TMPRSS2 and FUGW-MSPL containing TMPRSS2 and Vero/MSPL genes, (b) lentivirus envelope plasmids pMD2.G and psPAX2, (c) pseudotyped lentivirus particles produced in the HEK293 T cells, transduced to the Vero cells, and established in the cell lines by screening of the fluorescence expression.

A schematic diagram for the construction of Vero/TMPRSS2 and Vero/MSPL cell lines is shown in Figure 1. Briefly, human TMPRSS2 gene (BC051839) and MSPL gene (BC114928) were obtained from pDONR223 plasmids, kindly provided by Biogot Technology, Nanjing, China, and an HA tag sequence was added to the N-terminus of the TMPRSS2 and MSPL genes, and an enhanced green fluorescent protein (EGFP) sequence was added to the C-terminus of the TMPRSS2 and MSPL genes, giving rise to TMPRSS2-EGFP and MSPL-EGFP fusion genes. Subsequently, the cell lines were constructed by cloning the TMPRSS2 and MSPL genes into the lentiviral expression vector FUGW, giving rise to the recombinant plasmids FUGW-TMPRSS2 and FUGW-MSPL. FUGW control plasmids (without the target genes). The details of all the primers used in this study are listed in Table 1. All constructed plasmids were identified by PCR, enzyme digestion, and subsequent sequence analysis.

**Table 1.** Primers used in this study.

Target gene	Primer	Primer sequence (5ʹ→3ʹ)	Length
TMPRSS2 (BC051839)	F2-HA	GGAAGATCT^a^GCTAGCGCCACCATG***TACCCATACGATGTTCCAGATTACGCT***^b^GCTTTGAACTCAGGG	1524 bp
	R2-HA	GGCACCGGTGCCGTCTGCCCTCAT	
MSPL (BC114928)	F13-HA	CGCGGATCC^a^GCTAGCGCCACCATG***TACCCATACGATGTTCCAGATTACGCT***^b^GAGAGGGACAGCC	1734 bp
	R13-HA	GGCACCGGT^a^GGATTTTCTGAATCGC	
PEDV *N* (DQ072726)	PN-F	ACTGAGGGTGTTTTCTGGGTTGC	137 bp
	PN-R	GGTTCAACAATCTCAACTACACTGG	
β-actin (AB004047)	Beta-actin-F	AAGGATTCATATGTGGGCGATG	103 bp
	Beta-actin-R	TCTCCATGTCGTCCCAGTTGGT	
PEDV *S*	S-F	CCGGAATTC^a^ATGAAGTCTTTAACTTACTTCTGG	4152bp
	S-R	CTAGCTAGCTCGAG^a^TCACTGCACGTGGACCTTTTC	

^a.^Restriction enzyme recognition sites used for cloning are shown with underline;

^b.^HA tag is shown in bold and italic.

The third-generation lentiviral system, including plasmids FUGW (Addgene plasmid # 14,883), pMD2.G (Addgene plasmid # 12,259), and psPAX2 (Addgene plasmid # 12,260), were kind gifts from Dr Yandong Tang (Harbin Veterinary Research Institute, Chinese Academy of Agricultural Sciences, China). Human embryonic kidney 293 T cells (HEK293 T) were used to produce the pseudotyped lentivirus particles. In brief, HEK293 T cells in 100 mm×20 mm well dish were co-transfected with the three plasmids psPAX2 (4 μg), pMD2.G (2 μg), and FUGW-TMPRSS2/FUGW-MSPL/FUGW (6 μg) along with the Lipofectamine LTX & Plus Reagent (Invitrogen, Life Technologies, Carlsbad, CA, USA) according to the manufacturer’s protocol. After 6 h post-transfection, the cells were washed and cultivated in fresh DMEM containing 10% FBS, and then the supernatant samples containing lentivirus FUGW-TMPRSS2/FUGW-MSPL/FUGW were collected after 48 h and 72 h post-transfection [25], respectively.

Vero cells were transduced by lentivirus FUGW-TMPRSS2/FUGW-MSPL/FUGW according to the methods described previously with slight modifications [35]. Briefly, monolayers of Vero cells (1 × 10^5^ cells/well) in 24-well plates were incubated with lentivirus (multiplicity of infection, MOI = 1) diluted in DMEM (diluted at 1:100) containing 10% FBS and 8 μg/mL polybrene. After 12 h post-transduction, the media was replaced with fresh DMEM containing 10% FBS, and the cell status and the fluorescence expression were observed 72 h post-transduction. To generate Vero/TMPRSS2 and Vero/MSPL cell lines, cells were washed, trypsinized, and loaded onto an Aria II flow cytometer (BD Biosciences, San Jose, CA, USA) to screen the cell with green fluorescence [36], and then EGFP-positive cells were sorted and collected in 96-well cell plates at one cell per well, after two to three weeks, the single EGFP-positive colonies were observed by fluorescence microscope and the positive rate of EGFP was checked by flow cytometry according to methods described previously with slight modifications [37]. In this study, four consecutive FACsorts were performed until the positive rate of EGFP approached 100% [37].

### Expression of TMPRSS2 and MSPL

The expression of TMPRSS2 and MSPL in HEK293 T, Vero/TMPRSS2 and Vero/MSPL cell lines was verified by using reverse transcription PCR, fluorescence observation, and western blot assay. For reverse transcription PCR, total viral RNA of the cells was extracted using E.Z.N.A. Total RNA Kit I (Omega Bio-Tek, Doraville, GA, USA) and complementary DNA (cDNA) were generated with oligo(dT) primers using the Reverse Transcriptase Reagent Kit (Takara, Tokyo, Japan) in accordance with the manufacturer’s recommendations, and then, TMPRSS2 and MSPL genes were detected in HEK293 T, Vero/TMPRSS2 and Vero/MSPL cell lines with primers listed in Table 1. For the fluorescence observation, fluorescence images of HEK293 T 72 h post-transfection, and Vero/TMPRSS2 and Vero/MSPL cell lines after consecutively incubating 15 passages were acquired with the help of a fluorescence microscope (Leica, Wetzlar, Germany). For the western blot assay, HEK293 T 72 h post-transfection, and Vero/TMPRSS2 and Vero/MSPL cell lines after consecutively incubating 15 passages were washed with PBS, lysed with lysis buffer (Beyotime) containing phenylmethanesulfonyl fluoride (PMSF; Beyotime), and mixed with 5× sodium dodecyl sulfate (SDS) loading buffer. Then, proteins were then separated by sodium dodecyl sulfate-polyacrylamide gel electrophoresis (SDS-PAGE) and transferred onto the PVDF membranes (Millipore, Milford, MA, USA). The immunoblots were then developed using the mouse anti-HA monoclonal antibody (dilution at 1:4000, Sigma, USA) as the primary antibody, and horseradish peroxidase (HRP)-conjugated goat anti-mouse IgG antibody (dilution at 1:5000, Invitrogen, USA) as the secondary antibody. The results were visualized using a chemiluminescent substrate reagent (Thermo Scientific, Durham, NC) according to the manufacturer’s protocol and the expression of β-Actin was detected with an anti-β-actin antibody (Sigma, USA) as a loading control. In addition, Vero/TMPRSS2 and Vero/MSPL cell lines were further identified by FACS method as described above.

### Electron microscopy

The electron microscopic assay of PEDV LJB/03-infected Vero cells was performed as the method described previously [32]. Briefly, the PEDV LJB/03-infected Vero cells were fixed with 2.5% glutaraldehyde, and then post-fixed with 2% osmium tetroxide, dehydrated and embedded. Then, ultrathin sections were prepared and subsequently visualized with an electron microscope. Furthermore, for ultrastructural analysis, the supernatants of PEDV LJB/03-infected Vero cells were ultracentrifuged through a 25% sucrose cushion at 30,000 × g for 2 h at 4°C. Virions subsequently were resuspended and observed by transmission electron microscopy (H-7650, Hitachi, Tokyo, Japan).

### Real-time polymerase chain reaction (RT-PCR) analysis

To analyze the trypsin-dependence of PEDV LJB/03 P23 and PEDV isolates 2013-A and NJ, the copy number of viral RNA was determined by RT-PCR assay with SYBR Premix EX Taq II (Takara) and ABI 7500 real-time PCR system (Applied Biosystems, Carlsbad, CA, USA) according to the manufacturer’s instructions. The specific primers for amplifying the nucleocapsid gene of PEDV and the β-actin gene of Vero cells were used (Table 1). In brief, the total RNA of PEDV and complementary DNA (cDNA) were obtained as described above. The conditions of RT-PCR are stated as follows: 40 cycles of 30 s at 95°C, 3 s at 95°C, and 30 s at 60°C. Experiments were performed in triplicate and calculated by the 2−∆∆Ct method. The replication kinetics of intracellular PEDV LJB/03 P23 RNA in Vero cells (with or without trypsin), Vero/TMPRSS2 and Vero/MSPL stable cell lines were determined by RT-PCR assay. Briefly, the cells were seeded in 24-well plates and incubated with PEDV LJB/03 P23 at a multiplicity of infection (MOI) of 0.01 supplemented with 3 µg/mL trypsin (Vero cells) or an equivalent of PBS (Vero, Vero/TMPRSS2, and Vero/MSPL cells) for 1 h at 37°C, and Vero cells were mock-infected as the control. After washing, the samples were harvested at different time points post-infection and determined by RT-PCR as described above. To analyze the adaptation of PEDV isolates 2013-A and NJ in Vero/TMPRSS2 and Vero/MSPL cell lines, the small intestine tissue samples of PEDV-positive (PEDV 2013-A and NJ [32]) detected by reverse transcription PCR and PEDV colloidal gold Immune-chromatography assay were used, and the Vero cells, Vero/TMPRSS2, and Vero/MSPL cell lines were incubated with treated small intestine tissue samples containing PEDV 2013-A and NJ supplemented with 3 µg/mL trypsin or an equivalent of PBS for 72 h. After three serial passages, samples were harvested and determined by RT-PCR as described above.

### Viral plaque assay

In order to compare the infectivity of trypsin-dependent PEDV LJB/03 P23 in Vero cells (with or without trypsin), Vero/TMPRSS2 and Vero/MSPL cell lines, the viral titers were determined by the viral plaque assay. Briefly, the cell monolayers were incubated with PEDV LJB/03 P23 samples at a multiplicity of infection (MOI) of 0.1 supplemented with 3 µg/mL trypsin (Vero cells) or PBS (Vero cells, Vero/TMPRSS2, and Vero/MSPL cells). After 1 h of viral adsorption at 37°C, the inoculum was aspirated, the cells were washed twice with PBS and fixed with Minimum Essential Medium (MEM, Gibco) supplemented with 0.8% agarose. After incubation at 37°C and CPEs appeared, cells were stained with MEM supplemented with 0.01% Neutral Red Solution (Sigma). The syncytium was counted under a microscope as plaque and viral titer was expressed as PFU/mL.

### Comparison of S protein sequence before and after infection with PEDV

To determine whether the cell lines force any changes before and after infection with PEDV, PEDV LJB/03 S genes were sequenced and blasted after three serial passages. Briefly, PEDV LJB/03 was propagated (multiplicity of infection, MOI = 0.01) in the Vero, Vero/TMPRSS2, and Vero/MSPL cells; the total RNA of PEDV and complementary DNA (cDNA) before and after infection with PEDV were obtained as described above; and the PEDV LJB/03 S genes were amplified with S-F/S-R primer pairs (Table 1), sequenced and blasted.

### The cleavability of PEDV S protein in Vero, Vero/TMPRSS2, and Vero/MSPL cells

In order to confirm the cleavability of PEDV S protein in Vero cells (with or without 18 µg/mL trypsin), Vero/TMPRSS2 (without trypsin) and Vero/MSPL (without trypsin) cells, cells were seeded into six-well plates and transfected with 2 µg/well of PEDV-S plasmids (pCMV-HA-S encoding PEDV LJB/03 S protein with an N-terminal HA tag, kept in our laboratory) using the Lipofectamine LTX & Plus Reagent (Invitrogen, Life Technologies, Carlsbad, CA, USA), empty pCMV-HA plasmid as a negative control. The cells were collected, separated by sodium dodecyl sulfate-polyacrylamide gel electrophoresis (SDS-PAGE), and subsequently blotted onto PVDF membranes (Millipore, Milford, MA, USA). The PEDV S protein with an N-terminal HA-tag was analyzed using a mouse anti-HA monoclonal antibody (Sigma, USA) as the primary antibody and horseradish peroxidase (HRP)-conjugated goat anti-mouse IgG antibody (Sigma, USA) as the secondary antibody, and proteins were visualized using a chemiluminescent substrate reagent (Thermo Scientific, Durham, NC). The expression ofβ-Actin was detected with an anti-β-actin antibody (Sigma, USA) as a loading control.

### Observation of cytopathic effect

For determining the propagation of PEDV isolates, 2013-A and NJ in Vero cells, Vero/TMPRSS2 and Vero/MSPL stable cell lines, the cell monolayers were incubated with PEDV at an MOI = 0.01 supplemented with 3 µg/mL trypsin (Vero cells) or an equivalent of PBS (Vero, Vero/TMPRSS2 and Vero/MSPL cells) for 1 h at 37°C, and Vero cells were mock-infected as the control as described above, and then the PEDV-specific cytopathic effects (CPE) were observed under a microscope at 24, 48, and 72 h post-infection.

### Immunofluorescence assay

To compare the propagation effects of PEDV isolates 2013-A and NJ in Vero, Vero/TMPRSS2, and Vero/MSPL cells, an immunofluorescence assay was performed according to the methods described previously with slight modifications [38]. Vero/TMPRSS2, Vero/MSPL, and Vero cells were seeded in 12-well plates and incubated with PEDV isolates at MOI = 0.01 and cultured with or without trypsin as described above. After washing and fixing with 4% paraformaldehyde, and permeabilization with 0.2% Triton X-100, the cells were treated with rabbit anti-PEDV polyclonal antibody (dilution at 1:200) as primary antibody and TRITC-labeled goat anti-rabbit IgG (Sigma, St. Louis, MO, USA) as secondary antibody followed by the counterstain of nuclei with 4,6-diamidino-2-phenylindole (DAPI; Invitrogen, USA), fluorescence images were obtained by a fluorescence microscope (Leica, Wetzlar, Germany).

### Statistical analysis

Data are shown as the means ± standard errors of three replicates per test in a single experiment repeated in triplicate. Data were statistically analyzed by one-way ANOVA and Tukey’s multiple-comparison test, using GraphPad Prism v5.0 software. *P < 0.05, **P < 0.01, ***P < 0.001.

## Results

### Expression of TMPRSS2 and MSPL in 293 T Cells

**Figure 2.**
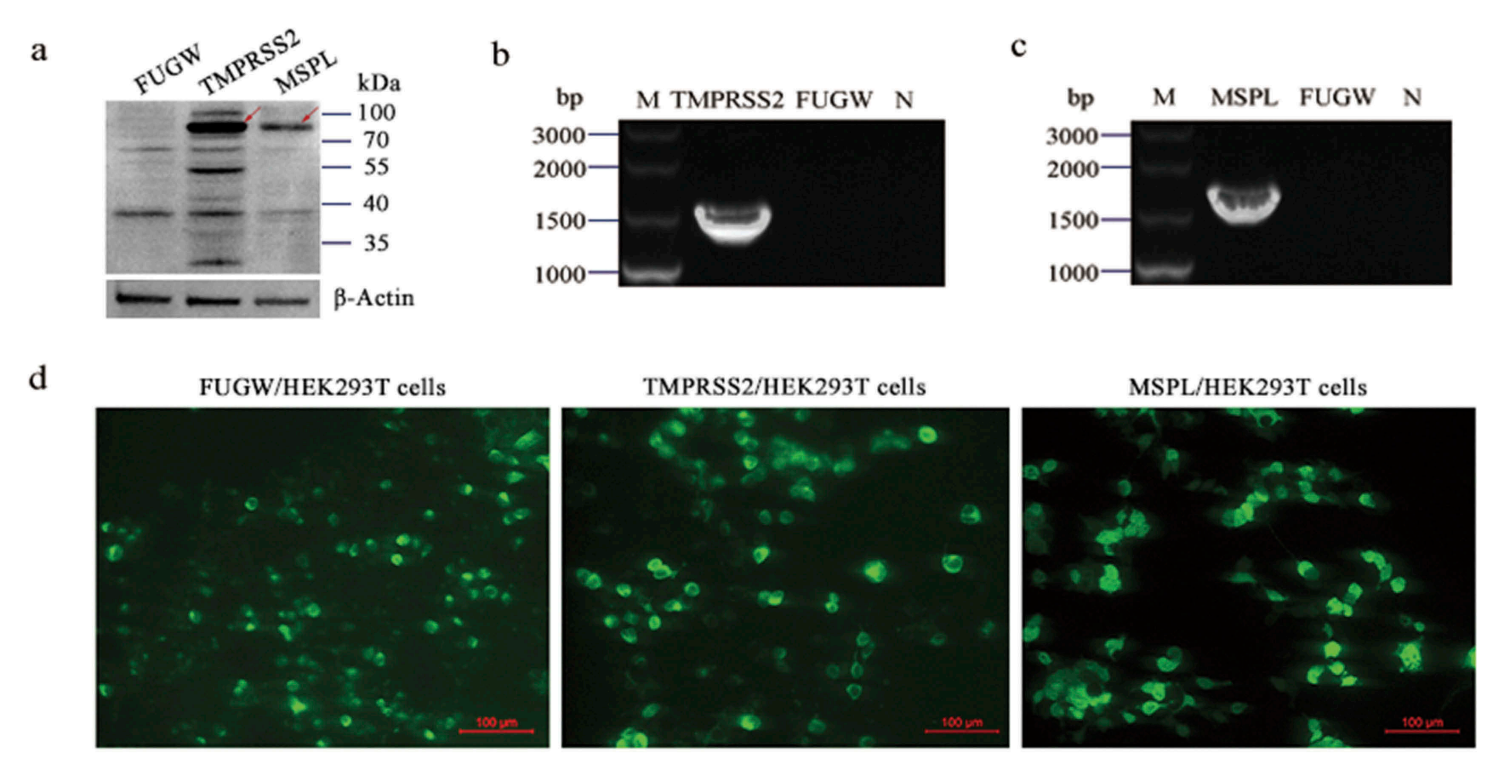
The expression of TMPRSS2 and MSPL genes in the HEK293 T cells. (a) The expression of TMPRSS2 and MSPL was detected by western blot with anti-HA monoclonal antibody after 72 h post-transfection. Immunoblot of TMPRSS2 and MSPL with the expected size was developed, but not for FUGW. FUGW: lentivirus FUGW in HEK293 T cells; TMPRSS2: lentivirus FUGW-TMPRSS2 in HEK293 T cells; MSPL: lentivirus FUGW-MSPL in HEK293 T cells. (b and c) TMPRSS2 and MSPL genes were detected by reverse transcription PCR. M: DNA Marker; N: negative control (water as negative control); FUGW: FUGW control plasmid without target genes. (d) Fluorescence images of FUGW/HEK293 T cells, TMPRSS2/HEK293 T cells, and MSPL/HEK293 T cells were observed after 72 h post-transfection.

The expression levels of TMPRSS2 and MSPL in HEK293 T cells were detected by fluorescence expression, reverse transcription PCR, and Western blot assay. As shown in Figure 2, the immunoblot band with the expected size was detected by western blot assay (a), and the specific reverse transcription PCR gene products for TMPRSS2 and MSPL were amplified (b and c), respectively, indicating that the TMPRSS2 and MSPL were efficiently expressed in HEK293 T cells. Moreover, specific green fluorescence was observed in FUGW-TMPRSS2, FUGW-MSPL, or FUGW groups by a fluorescence microscope (d), indicating that TMPRSS2-EGFP and MSPL-EGFP fusion proteins were expressed efficiently and lentivirus FUGW-TMPRSS2/FUGW-MSPL/FUGW were successfully produced in the HEK293 T cells.

### Verification of TMPRSS2 and MSPL in Vero Cells or stable cell Lines

**Figure 3.**
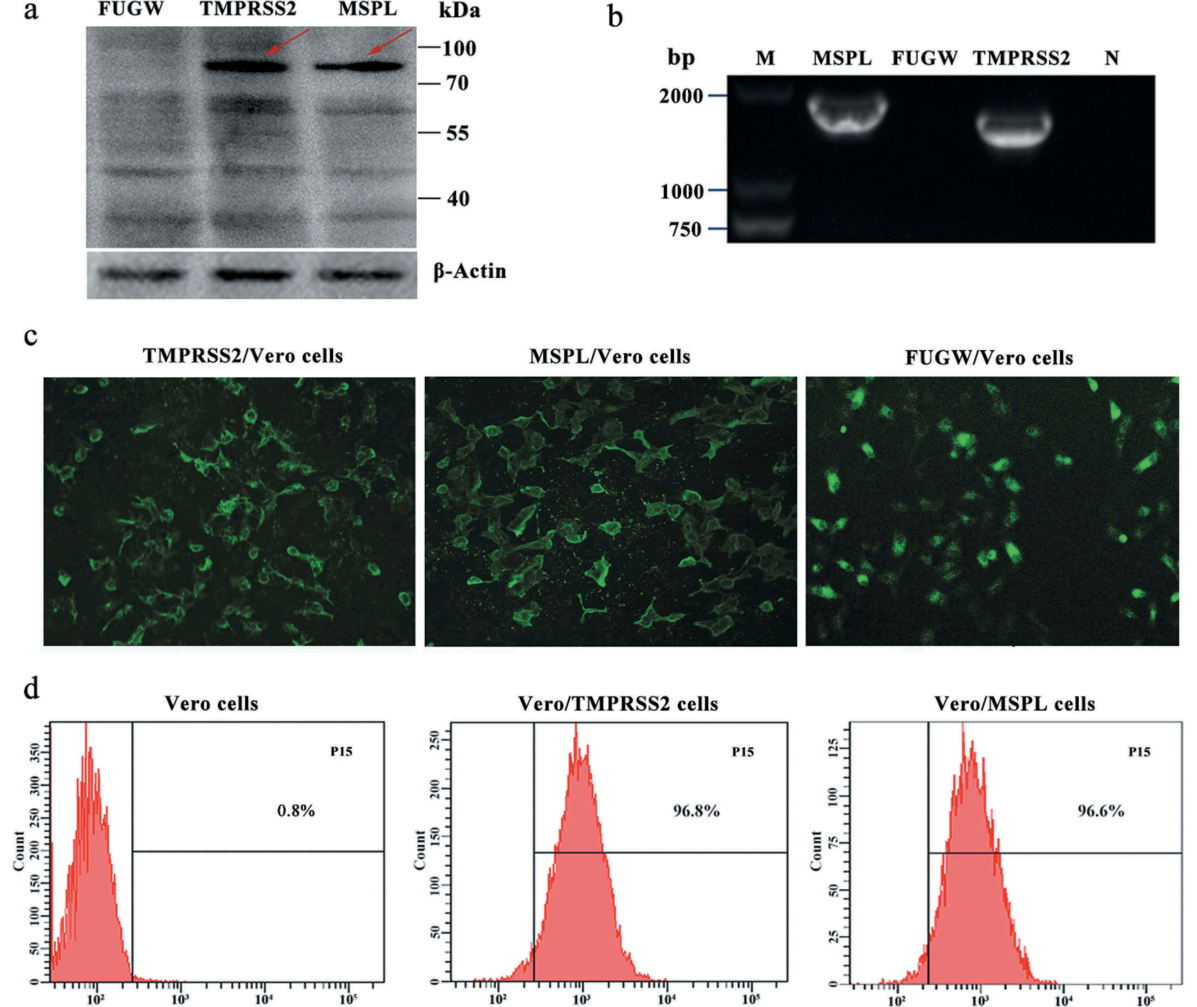
Stable expression of TMPRSS2 and MSPL genes in Vero cell lines. (a) The proteins in the lysates of Vero/TMPRSS2 and Vero/MSPL cell lines after consecutively incubating 15 passages were separated by SDS-PAGE, followed by identification by western blot with anti-HA monoclonal antibody. FUGW: FUGW/Vero cell lines; TMPRSS2: Vero/TMPRSS2 cell lines; MSPL: Vero/MSPL cell lines. (b) TMPRSS2 and MSPL genes were detected in Vero/TMPRSS2 and Vero/MSPL cell lines after consecutively incubating 15 passages by reverse transcription PCR. M: DNA Marker; N: negative control (water as negative control); FUGW: FUGW/Vero cell line. (c) Fluorescence images of Vero/TMPRSS2 and Vero/MSPL cell lines after consecutively incubating 15 passages were acquired by the fluorescence microscope, using FUGW/Vero cells as control. (d) Fluorescence intensity of Vero/TMPRSS2. Vero/MSPL and Vero cells were detected by flow cytometry after 15 serial passages.

Firstly, the endogenous presence of TMPRSS2 and MSPL genes in the Vero cells was detected by reverse transcription PCR assay with primers F2/R2-HA and F13/R13-HA as shown in Table 1, and the results were negative (Figure S2). Thereafter, in order to establish the Vero/TMPRSS2 and Vero/MSPL stable cell lines, Vero cells were transduced with lentivirus FUGW-TMPRSS2/FUGW-MSPL/FUGW produced from HEK293 T cells. The expression of TMPRSS2 and MSPL genes in Vero/TMPRSS2 and Vero/MSPL stable cell lines after consecutively incubating 15 passages was determined by the fluorescence expression, reverse transcription PCR assay, western blot assay with anti-HA antibody, and fluorescence-activated cell sorting (FACS). As shown in Figure 3, the TMPRSS2 and MSPL genes can be specifically amplified by reverse transcription PCR (b), indicating that the TMPRSS2 and MSPL genes were successfully recombined into the Vero cells. Subsequently, western blot assay (a) was performed to confirm the expression of TMPRSS2 and MSPL, and the results showed that the proteins of interest with the expected sizes (TMPRSS2: 82 kDa; MSPL: 89 kDa) were expressed in Vero/TMPRSS2 and Vero/MSPL stable cell lines, respectively, but not in the FUGW/Vero cell line. At the same time, fluorescence was observed on the surface of Vero/TMPRSS2, Vero/MSPL, and FUGW/Vero stable cell lines (c), finally, in order to confirm the stability of the Vero/TMPRSS2 and Vero/MSPL cell lines, we performed FACS assay to examine the fluorescence intensity of the two Vero cell lines after 15 serial passages, and the results showed that the EGFP-positive rate of Vero/TMPRSS2 and Vero/MSPL cell lines was approximately 100% (d), indicating that Vero/TMPRSS2 and Vero/MSPL stable cell lines were successfully established.

### The propagation of cell-adapted PEDV strain LJB/03 P23 in Vero cells (with or without trypsin), Vero/TMPRSS2, and Vero/MSPL cells

**Figure 4.**
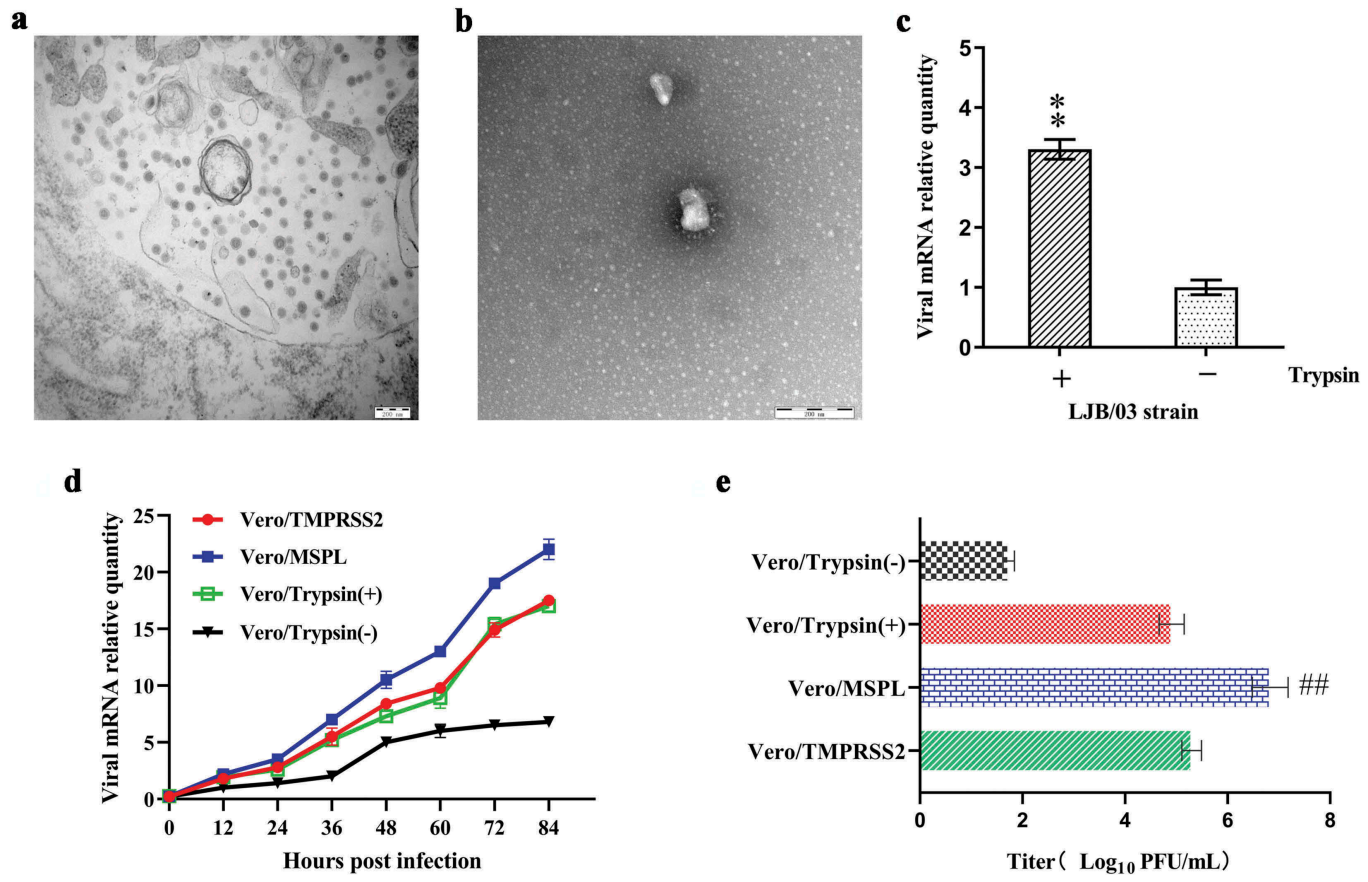
The propagation of cell-adapted PEDV strain LJB/03 P23 in Vero cells (with or without trypsin), Vero/TMPRSS2 and Vero/MSPL cells. Ultrathin sections of PEDV LJB/03-infected Vero cells at 24 h post-infection were prepared, and massive virus particles as shown by the arrow (Bar = 200 nm) were observed by the electron microscopy (a); PEDV LJB/03 particles in culture media as shown by the arrow (Bar = 200 nm) were observed by the transmission electron microscopy (b). (c) Trypsin-dependence of LJB/03 P23 was determined by RT-PCR assay with SYBR Premix EX Taq II (* p < 0.05, ** p < 0.01 compared to the Vero cells without trypsin group). (d) Replication kinetics of PEDV LJB/03 P23 in Vero cells (with or without trypsin), Vero/TMPRSS2, and Vero/MSPL cells were determined by RT-PCR, respectively. Vero cells (with 3 µg/mL or without trypsin), Vero/TMPRSS2, and Vero/MSPL cells were incubated with PEDV LJB/03 P23 at MOI = 0.01 and samples were harvested at 0, 12, 24, 36, 48, 60, 72, and 84 h post-infection, and then Viral RNA copy numbers were determined by RT-PCR. (e) PEDV LJB/03 P23 titers in Vero cells (with or without trypsin), Vero/TMPRSS2, and Vero/MSPL cells were determined by the plaque assay. Cells were incubated with PEDV LJB/03 P23 at MOI = 0.1 supplemented with 3 µg/mL trypsin (Vero cells) or PBS (Vero cells, Vero/TMPRSS2 and Vero/MSPL cells) and the viral titers (PFU/mL) were calculated by counting the syncytias. #P < 0.05, ##P < 0.01 as compared to Vero/TMPRSS2 cells.

In order to confirm the ultra-structure and trypsin dependence of PEDV LJB/03 P23, the electron microscopy and RT-PCR assay were performed according to the methods described above. The result of electron microscopy assay for PEDV LJB/03-infected Vero cells revealed that massive virus particles with a dense core accumulated in the cytoplasm post-infection (Figure 4(a)), and the mature particles were circular with a diameter of 80–120 nm, characterized by bulbous projections of coronaviruses on the surface (Figure 4(b)). To confirm the trypsin-dependence of PEDV LJB/03 P23, the viral mRNA levels after viral adsorption were detected by RT-PCR assay, and the results revealed that the relative RNA levels of PEDV propagated in the Vero cells supplemented with trypsin were significantly higher than that without trypsin (Figure 4(c)), indicating that PEDV LJB/03 P23 was trypsin-dependent strain.

To compare the propagation effect of PEDV LJB/03 P23 on the Vero cells (with or without trypsin), Vero/TMPRSS2, and Vero/MSPL cells, the replication kinetics, and viral titration were determined by RT-PCR and plaque assay, respectively. The results of replication kinetics demonstrated that the RNA levels of PEDV LJB/03 P23 propagated on Vero/TMPRSS2, Vero/MSPL, and Vero cells (with trypsin) were substantially higher than that propagated on the Vero cells without trypsin (Figure 4(d)). Moreover, the RNA levels of PEDV markedly increased in the Vero/MSPL cells as compared to the Vero/TMPRSS2 cells. Nevertheless, there was no significant difference in the RNA levels between the Vero cells with trypsin and Vero/TMPRSS2 cells. Our data indicated that TMPRSS2 and MSPL support the propagation of cell-adapted PEDV LJB/03. Between the two of them, the effect of MSPL was better than that of TMPRSS2 and trypsin. Subsequently, in order to further compare the replication ability of PEDV LJB/03 P23 in Vero cells (with or without trypsin), Vero/TMPRSS2, and Vero/MSPL cell lines, the viral titration was determined by plaque assay with Neutral Red Solution. The results showed that the PEDV titration in the Vero/MSPL cells was significantly higher than that in the Vero cells with trypsin and Vero/TMPRSS2 cells (Figure 4(e)). Nevertheless, there was no notable difference in the viral titration observed between Vero cells with trypsin group and Vero/TMPRSS2 cell group (Figure 4(e)). The Vero/TMPRSS2 cells and Vero/MSPL cells can effectively enhance the titration of cell-adapted and trypsin-dependent PEDV in the absence of trypsin supplementation, and between the two of them, Vero/MSPL cell is better. Our results indicated that the effect of MSPL promoting replication and propagation of cell-adapted and trypsin-dependent PEDV was significantly better than that of trypsin and TMPRSS2, suggesting a promising tool for the propagation of PEDV in large-scale industrial vaccine production.

### Comparison of S protein sequence before and after infection with PEDV LJB/03 P23

To determine whether the cell lines force any changes of PEDV before and after infected Vero, Vero/TMPRSS2 and Vero/MSPL cells, PEDV LJB/03 S gene was amplified, sequenced and blasted after three serial passages. The results demonstrated that there was no change in nucleotide and protein sequences of S gene before and after exposure (Figure S1), indicating that Vero/TMPRSS2, and Vero/MSPL cell lines, like Vero cells, can be used for propagating PEDV without any force changes.

### Proteolytic activation of PEDV S protein in Vero, Vero/TMPRSS2, and Vero/MSPL cells

**Figure 5.**
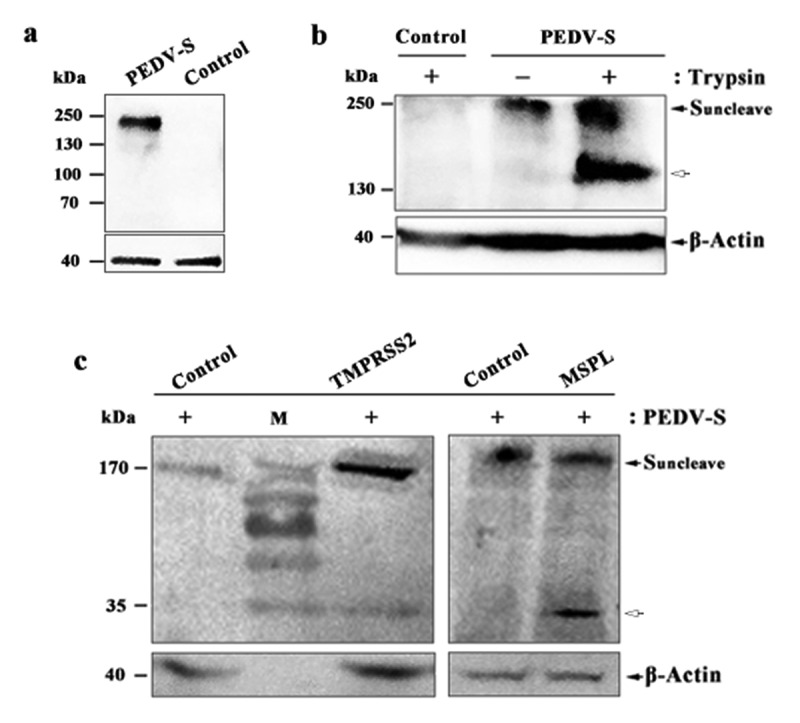
Proteolytic activation of PEDV S protein in Vero, Vero/TMPRSS2 and Vero/MSPL cells. (a) The expression of PEDV S genes in the Vero cells. Vero cells were transfected with PEDV-S plasmids (pCMV-HA-S encoding PEDV LJB/03 S protein with a HA tag) and the expression of PEDV S genes in the Vero cells were detected by western blot with anti-HA monoclonal antibody after 72 h post-transfection. Empty pCMV-HA plasmid as control. (b and c) Cleavage of PEDV S protein in Vero cells (with or with trypsin), Vero/TMPRSS2 and Vero/MSPL cells. Arrows indicate either uncleaved S protein (black arrows) or N-terminal cleavage S protein products (white arrow). Empty pCMV-HA plasmid as control.

To analyze the proteolytic activation of PEDV S protein in Vero cells (with or without trypsin), Vero/TMPRSS2 (without trypsin), and Vero/MSPL (without trypsin) cells, the cleavability of S protein was detected by western blot. Western blot analysis showed that an expected immunoreactive band of S protein (200 kDa) was detected in PEDV-S plasmid group, but not in empty plasmid group (Figure 5(a)). Furthermore, PEDV S protein can be cleaved by trypsin in Vero cells, compared to empty plasmid control and cells without trypsin supplementation (Figure 5(b)). The results demonstrated that the full-length of S protein (200 kDa in size, black arrows) was detected in Vero cells (with or without trypsin), furthermore, the cleavage products of S protein (150 kDa in size, white arrow) were observed (Figure 5(b)). We next sought to confirm the cleavability of PEDV S protein in Vero/TMPRSS2 and Vero/MSPL cells by western blot. The results indicated that the full-length of S protein (200 kDa in size, black arrows) can be cleaved in Vero/TMPRSS2 and Vero/MSPL cells with the same 35kDa cleavage fragments (Figure 5(c), white arrow), indicating that TMPRSS2 and MSPL, constitutively expressing in Vero cells, are responsible for the cleavage and activation of PEDV S protein, which may be correlated with the ability to support the replication of PEDV.

### Trypsin-dependence and propagation of PEDV isolates in Vero/TMPRSS2 and Vero/MSPL cells

**Figure 6.**
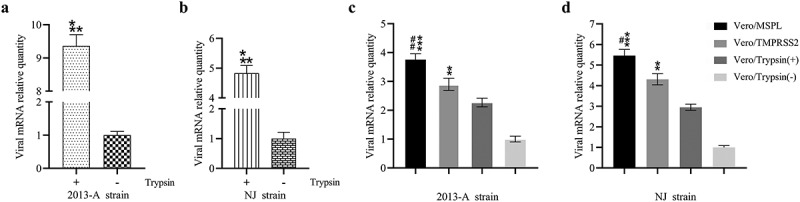
Trypsin-dependence and propagation of PEDV isolates 2013-A and NJ in Vero/TMPRSS2, Vero/MSPL cells and Vero cells (with or without trypsin). (a and b) Trypsin-dependence of 2013-A and NJ were determined by RT-PCR assay with the 2^−∆∆Ct^ method as described above. Error bars indicate the standard error of three independent experiments and the viral mRNA relative quantity of the Trypsin(-) group set to 1. (* p < 0.05, ** p < 0.01,*** p < 0.001 compared to the Vero cells without trypsin). (c and d) The propagation of PEDV isolates 2013-A and NJ in Vero/TMPRSS2, Vero/MSPL, cells and Vero cells (with 3 µg/mL or without trypsin) was analyzed by RT-PCR at 72 h post-infection. The viral mRNA relative quantity of the Vero/Trypsin(-) set to 1 and Error bars indicate the standard error of the mean. (*P < 0.05, **P < 0.01 *** p < 0.001 compared to the Vero cells with trypsin; #P < 0.05, ##P < 0.01 as compared to Vero/TMPRSS2 cells).

To confirm whether Vero/TMPRSS2 and Vero/MSPL cells can effectively propagate PEDV isolates without trypsin, we explored the trypsin-dependence, replication and propagation ability of PEDV isolates 2013-A and NJ by RT-PCR assay (Figure 6). The result of trypsin-dependence of PEDV isolates revealed that the viral RNA levels in 2013-A-infected and NJ-infected Vero cells with trypsin were almost 9-fold and 5-fold higher than that in the Vero cells in absence of trypsin, respectively, indicating that the PEDV isolates 2013-A and NJ were trypsin-dependent viruses (a and b). Moreover, Vero/TMPRSS2 and Vero/MSPL cells (without trypsin) or Vero cells (with or without 3 µg/mL trypsin) were incubated with treated clinical intestine tissue samples containing PEDV 2013-A and NJ followed by detection by RT-PCR, and the results showed that MSPL and TMPRSS2 could effectively facilitate 2013-A (c) and NJ replication (d) at 72 h post-infection compared to the trypsin treatment group, and the effect of MSPL was the most significant. Our results indicated that using Vero/TMPRSS2 and Vero/MSPL cells can effectively promote propagation of PEDV isolates in the absence of exogenous trypsin, which is superior to 3 µg/mL trypsin, suggesting these cells as useful tool for the isolation of PEDV from the clinical samples.

### Adaptation of PEDV isolates to Vero/TMPRSS2 and Vero/MSPL stable cell lines

**Figure 7.**
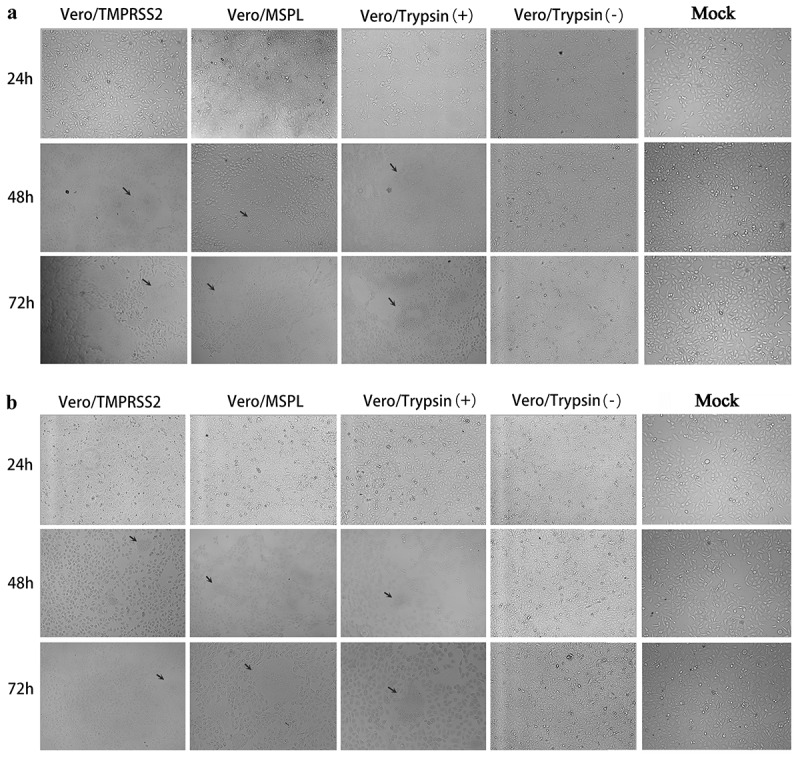
The cytopathic effects (CPEs) of Vero/TMPRSS2, Vero/MSPL, and Vero cells (without or with 3 μg/mL trypsin) incubated with PEDV isolates 2013-A and NJ. The Vero/TMPRSS2, Vero/MSPL, and Vero cells (without or with 3 μg/mL trypsin) were, respectively, incubated with PEDV isolates (2013-A and NJ) at an MOI = 0.01, followed by observation of CPEs. (a) CPEs observation of trypsin-dependent PEDV 2013-A at 24, 48, and 72 h post-infection; (b) CPEs observation of trypsin-dependent PEDV NJ at 24, 48, and 72 h post-infection. Arrows indicate syncytium.

**Figure 8.**
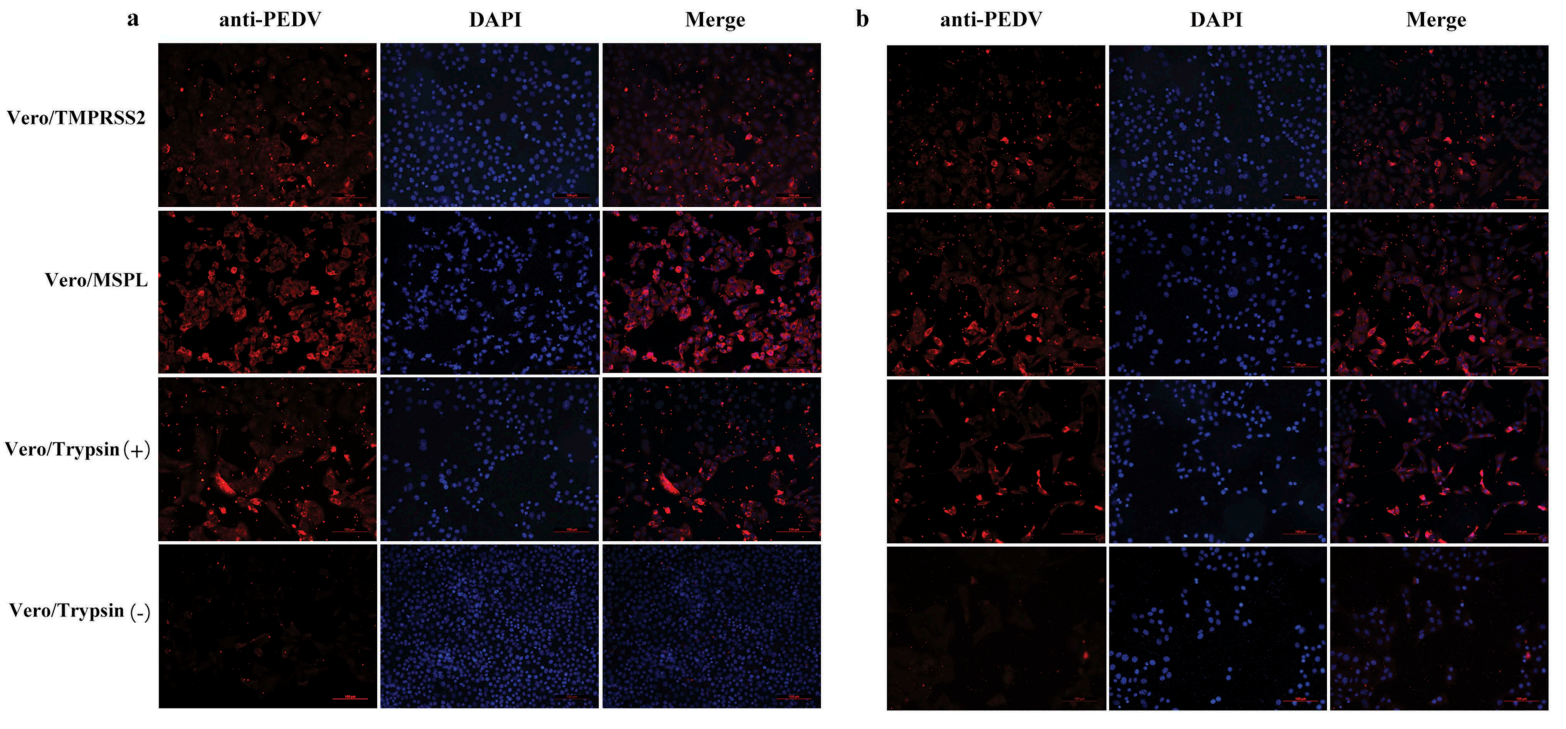
Immunofluorescence of Vero/TMPRSS2, Vero/MSPL, and Vero cells (without or with 3 μg/mL trypsin) infected by PEDV isolates 2013-A and NJ at an MOI = 0.01. (a) The fluorescence intensity was observed in trypsin-dependent PEDV 2013-A at 48 h post-infection. (b) The fluorescence intensity of trypsin-dependent PEDV NJ at 48 h post-infection.

To evaluate the promotion effects of Vero/TMPRSS2 and Vero/MSPL cell lines on PEDV isolates, the cytopathic effects (CPE) of PEDV isolates 2013-A and NJ propagated in Vero/TMPRSS2, Vero/MSPL, and Vero cells (without or with 3ug/mL trypsin) were observed at 24, 48, and 72 h post-infection as described above. As shown in Figure 7, the cytopathic trend of PEDV isolates 2013-A (a) and NJ (b) was the same at different time points. No obvious CPEs were observed in all cells at 24 h post-infection. The CPEs observed on Vero/TMPRSS2, Vero/MSPL, and Vero cells (with 3 µg/mL trypsin) were significantly higher than those on Vero cells without trypsin and mock-infected cells, and Vero/TMPRSS2 and Vero/MSPL cells showed a more typical CPE than the Vero cells with or without trypsin (3 µg/mL) post-infection, including cell rounding, multinucleated syncytia formation, shrinkage, and abscission. Moreover, Vero/MSPL cells developed more significant and severe cytopathy than the Vero/TMPRSS2 cells at 72 h post-infection. Syncytium was observed in Vero/TMPRSS2, Vero/MSPL, and Vero cells (with 3 µg/mL trypsin), while no syncytium formation was seen in Vero cells (without trypsin) and mock-infected cells. Furthermore, after PEDV isolates 2013-A and NJ infection, syncytium (cell-cell fusion) in Vero/TMPRSS2, Vero/MSPL and Vero cells adding trypsin expanded with time, suggesting that TMPRSS2 and MSPL contribute to the PEDV infection. Collectively, trypsin-dependent PEDV isolates 2013-A and NJ could effectively replicate in the Vero/TMPRSS2 and Vero/MSPL cells without trypsin, indicating that the Vero/TMPRSS2, especially Vero/MSPL cells, can contribute to the propagation and infection of PEDV isolates without trypsin supplementation. In parallel, immunofluorescence assay was performed following the same protocol as described above, to identify the PEDV isolates (2013-A and NJ) propagated in Vero/TMPRSS2 and Vero/MSPL cells without trypsin, and in the Vero cells with or without trypsin at 48 h post-infection. As shown in Figure 8, specific red fluorescence was observed in Vero/TMPRSS2, Vero/MSPL, and the Vero cells with trypsin infected with PEDV 2013-A (a) and PEDV NJ (b), and more fluorescence was observed in the Vero/MSPL cells, while lesser fluorescence was observed in the Vero cells without trypsin, indicating that trypsinase-dependent PEDV isolates can be propagated in Vero/TMPRSS2, particularly Vero/MSPL cells, without any additional trypsin addition.

## Discussion

Porcine epidemic diarrhea (PED) is an acute, highly contagious, deadly enteric disease in the piglets [39], characterized by watery diarrhea, dehydration, vomiting, with severe mortality in the neonatal piglets [40]. PED reemerged in China since 2010 [41] and in the USA since 2013 [5], and it resulted in substantial economic losses to the pig industry worldwide. Undoubtedly, vaccines are crucial for the effective prevention and control of PED epidemics. Nevertheless, PEDV undergoes frequent variation, recombination, and evolution, resulting in reemergence of virulent PEDV and vaccination failure [8,15,42–44]. Therefore, the establishment of effective isolation and culture methods for PEDV is urgently necessary to further study the virus biology, immunoassays, and defense against viral infection, due to separating PEDV is the first step.

PEDV propagation *in vitro* requires trypsin in order to activate its S protein [18]. However, previous studies have revealed that exogenous trypsin can damage the host cells and viral infectivity of the PEDV isolates maybe be lost following serial passages in cell culture supplemented with trypsin [19]. Additionally, mutational and evolutional PEDV are insensitive to trypsin [22]. In this study, we determined the trypsin dependence of cell-adapted PEDV strain LJB/03 P23 and P146 (after 23 or 146 serial passages in cell cultures), and the results showed that there were no significant differences in relative levels of viral RNA of LJB/03 P146 in the Vero cells with or without trypsin, while LJB/03 P23 showed obvious trypsin dependence. The sequence alignment analysis of the S protein showed that the S protein of LJB/03 P146 harbors a mutation from R to G in the 890th amino acid position (results not shown), in accordance with the previous reports in PEDV DR13, which presumably affected PEDV’s dependence on trypsin [22,45]. Therefore, the above results demonstrated that PEDV has incomplete trypsin-dependent properties with serial passages *in vitro*. Therefore, we selected trypsin-dependent LJB/03 P23 for subsequent research, in order to find alternatives to trypsin for culturing PEDV *in vitro*.

Recently, Shi *et al*. successfully utilized porcine IECs to isolate and propagate PEDV replacing Vero cells, suggesting that trypsin-like proteases play a critical role in facilitating the propagation of PEDV in IECs [32]. Additionally, previous studies have reported that trypsin-like serine proteases such as type II transmembrane serine protease 2 (TMPRSS2) and MSPL may facilitate the replication and spread of human influenza viruses, SARS-CoV, HMPV, and MERS-CoV in the absence of trypsin [26,27]. Moreover, TMPRSS2 that was stably expressed in the Vero cells could facilitate the release of viruses from the infected Vero cells [18]. Nevertheless, it remains unknown whether the stable Vero cell lines expressing TMPRSS2 and MSPL can be used for the isolation and culture of PEDV isolates. In this study, Vero cell lines constitutively expressing TMPRSS2 (Vero/TMPRSS2 cells) and MSPL (Vero/MSPL cells) were established with the third-generation lentivirus system including FUGW, pMD2.G, and psPAX2 plasmids followed by evaluation with cell-adapted PEDV strain and PEDV isolates. Du *et al*. utilized lentivirus system to construct a stable nonbovine CRL cell line expressing an intron-encoding endonuclease I-SceI [35], which can integrate target genes into the genome of host cells including dividing and nondividing cells [46], thereby achieving long-term, efficient, and stable expression [47].

In order to obtain the target genes, we attempted to amplify the target genes encoding the TMPRSS2 and MSPL of porcine origin from bronchus, lung, trachea, and small intestine tissues of pig, according to the predicted sequences of swine published in NCBI, in accordance with the previous report, but failed [25]. Therefore, TMPRSS2 and MSPL of the human origin were used for establishing the stable Vero cell lines in this study. Moreover, the presence of genes encoding TMPRSS2 and MSPL in the Vero cells was determined, and results indicated that there may be no endogenous genes encoding TMPRSS2 and MSPL in the Vero cells, or the expression level of these genes may be low or limited [48]. Therefore, it is feasible and significant to study the effect of TMPRSS2 and MSPL on PEDV propagation in Vero cells. HEK293 T cells, owing to tolerate toxic, fusogenic, or cytostatic vector and instantaneous production speed [49], were used to produce pseudotyped lentivirus particles with strong infection ability, good security, and stability. Furthermore, Vero cell has stable genetic characteristics, toleration for exogenous trypsin [50], and it supports the growth of various trypsin-dependent viruses including influenza virus [51], PEDV [18], hMPV [52], SARS [53], rotavirus [54], HCoV-229E [55], and MHV [56] etc., which was applied in this study to generate cell lines stably expressing TMPRSS2 and MSPL. The successful construction of Vero/TMPRSS2 and Vero/MSPL cell lines constitutively expressing TMPRSS2 and MSPL, respectively, would provide a useful tool for the propagation of trypsin-dependent viruses, simplification of operation, theoretical study of viral molecules, and large-scale production of biological products.

To exclude the effect of residual plasmids with target genes into Vero cells [57] and to prove the stability of TMPRSS2 and MSPL expression in Vero cells, Vero/TMPRSS2 and Vero/MSPL cell lines were consecutively incubated for 15 passages, followed by the identification the respective gene by reverse transcription PCR, EGFP fluorescence observation, and western blot. Western blot results showed that target bands were examined but the immunoreactive bands are not unique, which is consistent with previous work [26]. We speculated that serine proteases TMPRSS2 and MSPL, the glycosylated proteins, showed multiple cut bands with different sizes by their own proteolytic activity [58]. Moreover, the results indicated that the bands, larger than the target bands (TMPRSS2,82 kDa; MSPL, 89 kDa), were detected in this study, and we speculated that caused by phosphorylation [58]. Additionally, TMPRSS2 and MSPL, type II transmembrane serine proteases favorable for activation of viral surface proteins, were examined on the plasma membrane of cells in this study (Figures 2d and 3c) in accordance with the previous results [23,24,59]. The green fluorescence signal of Vero/TMPRSS2 and Vero/MSPL cells approached approximately 100% positive after incubating 15 passages analyzed by flow cytometry, confirming that the stable Vero cell lines were successfully established [36] and can be used for propagating PEDV without any force changes like Vero cells.

In this study, the propagation of cell-adapted PEDV strain LJB/03 P23 was tested using Vero cells (with or without trypsin), Vero/TMPRSS2, and Vero/MSPL cells. Initially, to confirm the existence of the virus, PEDV LJB/03 P23 was identified by an electron microscopic technique, characterized by a dense core and bulbous projections of coronaviruses on the surface. Further, to explore the propagation in Vero cells (with or without trypsin), replication kinetics and titers of trypsin-dependent PEDV LJB/03 P23 were determined by RT-PCR and plaque assay, respectively. The results showed that the ability of TMPRSS2 and MSPL, especially MSPL, to promote replication of trypsin-dependent PEDV was significantly stronger than that of trypsin. Recent advances in research demonstrated that TMPRSS13 (MSPL) owns the largest intracellular domain of all the members of the type II transmembrane serine protease (TTSP) family, and was subjected to post-translational modifications due to its richness in proline, serine, and threonine residues, which can enhance the function of a protein’s existence in the cells [60], in accordance with our results the ability of MSPL to enhance cell-adapted PEDV strain LJB/03 P23 proliferation was obviously superior to TMPRSS2. Therefore, the above observations demonstrated that TMPRSS2, especially MSPL, can replace trypsin for the cell-adapted PEDV culture in vitro, indicating great application prospect in the PEDV industrial vaccine production.

Previous studies have demonstrated that TMPRSS2 support multicycle replication of inﬂuenza virus and human metapneumovirus by activating HA protein and fusion protein F, respectively [23,26]. Alternatively, Shi *et al*. revealed that MSPL and TMPRSS2 could cleave and activate PEDV S protein by cotransfecting PEDV S plasmids and MSPL or TMPRSS2-expressing plasmids into Vero cells [25]. In this study, to confirm the proteolytic activation of trypsin, TMPRSS2, and MSPL on PEDV S protein, PEDV S plasmids were transfected in Vero cells (with or without trypsin), Vero/TMPRSS2 (without trypsin) and Vero/MSPL (without trypsin) cells and the cleavability of S protein were detected by western blot. The results showed that PEDV S protein was expressed and cleaved by trypsin; the PEDV S protein (with a N-terminal HA tag) full-length showed 200 kDa in size due to glycosylation modification [18,22] and the cleavage products showed 150 kDa in size which confirmed 50 kDa cleavage products (with a C-terminal Flag tag) in previous study [22], suggesting the same cleavage site of trypsin. Nevertheless, the cleavability of S protein in Vero/TMPRSS2 and Vero/MSPL cells showed approximately 35kDa cleavage fragments (N-terminal fraction), which confirmed 160 kDa cleavage products (C-terminal fraction) in the previous research [18], due to the full-length of PEDV S protein was about 200 kDa. Shi *et al*. investigated the co-localization of proteases (TMPRSS2 and MSPL) and PEDV S protein, and confirmed the roles of proteases (TMPRSS2 and MSPL) in the PEDV S protein activation, indicating that MSPL and TMPRSS2 may interact with PEDV S protein to activate PEDV entry, which may be the way to cleave S protein by MSPL and TMPRSS2 [25]. Collectively, these observations suggested that TMPRSS2 and MSPL contribute to the PEDV S protein activation in Vero/TMPRSS2 and Vero/MSPL cells without trypsin supplementation, which may be correlated with the ability to support the entry and propagation of PEDV, but the specific mechanism needs to be further investigated.

Subsequently, we used two PEDV isolates 2013-A and NJ to evaluate whether the established Vero/TMPRSS2 and Vero/MSPL cell lines can be used for the isolation and culture of PEDV from clinical tissue samples. The RT-PCR results suggested that the PEDV isolates (2013-A and NJ) were trypsin-dependent virus and the propagation abilities in Vero/TMPRSS2 cells were slightly higher than that in the Vero cells treated with trypsin 72 h post-transfection, indicating different trypsin concentrations might account for the differences in results since Shirato *et al*. showed the Vero/TMPRSS2 cell was significantly different from 1.25 µg/mL trypsin [18]. Significantly, the Vero/MSPL cells were more effective in facilitating 2013-A and NJ propagation than the Vero/TMPRSS2 cells and Vero cells with 3 µg/mL of exogenous trypsin, mainly due to the characteristic structure of MSPL mentioned above. Furthermore, compared to all the cell groups infected with the PEDV isolate strain NJ, the viral mRNA levels were statistically higher in the PEDV isolate strain 2013-A group, indicating that the isolation and propagation of different PEDV strains showed difference, although they were in the same cell lines.

Shi *et al*. propagated porcine epidemic diarrhea virus (PEDV) strain NJ supplemented with 5 µg/mL trypsin [32]; Wicht *et al*. propagated and titrated PEDV strain CV777 containing 15 µg/ml trypsin to investigate the impact of trypsin on PEDV S [22], and Shirato *et al*. propagated PEDV strain MK in Vero cells adding 2.5 µg/ml of trypsin and compared the effect of proteases (trypsin and TMPRSS2) on PEDV MK strain infection with 1.25 µg/ml of trypsin [18], indicating the different PEDV strains might account for the differences in trypsin concentrations. However, PEDV LJB/03, used in this study, was isolated and propagated in our laboratory, and was proved to be suitable for propagation with trypsin at a concentration of 3 µg/ml [25,34]. Furthermore, the concentration of trypsin in propagating PEDV isolates 2013-A and NJ was determined in this study, and the results showed that PEDV isolates were also suitable for propagation with trypsin at a concentration of 3 µg/ml (data not shown). Therefore, in this study, we compared the propagation of PEDV LJB/03 and PEDV isolates in Vero/TMPRSS2, Vero/MSPL with Vero cells supplemented with 3 µg/mL trypsin, and results showed that the promoting effects of Vero/MSPL and Vero/TMPRSS2 cells on the propagation of PEDV isolates were superior to 3 μg/mL trypsin, which may be depend on PEDV strains and the dose of trypsin (3 μg/mL) used in this study.

Furthermore, we compared the promotion effect of Vero/TMPRSS2 and Vero/MSPL cell lines on PEDV isolates (2013-A and NJ) by detecting cytopathic effects and fluorescence intensity post-infection. Collectively, trypsin-dependent PEDV isolates 2013-A and NJ can replicate in Vero/TMPRSS2 and Vero/MSPL cells without exogenous trypsin addition, and the Vero/MSPL cells developed more severe CPEs and more cell exfoliation than the Vero/TMPRSS2 and Vero cells with trypsin at 72 h post-infection, indicating that the stable Vero cell lines, especially Vero/MSPL cell lines, contribute to the propagation of PEDV isolates without trypsin supplementation. In order to observe the proliferation of PEDV more intuitively and clearly, the syncytium formation (cell-cell fusion), the characteristic CPE [61], were observed post-infection, and our results clearly demonstrated that cell-cell fusion was shown in Vero/TMPRSS2, Vero/MSPL, and Vero cells with 3 ug/mL trypsin, but not in the Vero cells without trypsin. The results were similar to those reported by Shi *et al*., who utilized cell transfection but not cell lines [25]. Previous studies have reported that TMPRSS2 and MSPL played a key role in the release of PEDV particles on cells after replication occurs and facilitated the PEDV replication by promoting cell–cell fusion and virus–cell fusion [18,25]. In this study, the syncytium formation (cell-cell fusion) was observed clearly in Vero/TMPRSS2, Vero/MSPL cell lines stably expressing TMPRSS2 and MSPL post-infection, which most likely promote the enhancement of PEDV infection. In addition, it is generally recognized that trypsin facilitates PEDV infection in Vero cells during the different stages of the virus life cycle [62,63], demonstrating that the endogenous TMPRSS2 and MSPL in the cell lines play a crucial role in viral infection and replication by facilitating cell–cell fusion, but the specific mechanisms remain to be clarified.

In conclusion, Vero/TMPRSS2 and Vero/MSPL cell lines stably expressing TMPRSS2 and MSPL, respectively, were established with lentivirus system in this study. The results clearly demonstrated that Vero/TMPRSS2, especially Vero/MSPL, can replace exogenous trypsin for culturing cell-adapted PEDV strains in vitro, effectively facilitate the isolation of PEDV from clinical samples, significantly enhance the titer of PEDV and promote viral propagation by activating PEDV S protein and cell–cell fusion, suggesting a promising approach for isolating and propagating PEDV and even other trypsin-dependent viruses in vitro without trypsin treatment.

## Supplementary Material

Supplemental Material
