# Supplementary material for: Establishment of stable Vero cell lines expressing TMPRSS2 and MSPL: A useful tool for propagating porcine epidemic diarrhea virus in the absence of exogenous trypsin
Source: Virulence. 2020 May 29;11(1):669–85. doi: 10.1080/21505594.2020.1770491 (PMC7550007; doi:10.1080/21505594.2020.1770491)
Supplement: Supplemental Material [file KVIR_A_1770491_SM8718.zip › Suppl captions.docx]

**Fig. S1** Comparison of S protein sequence before and after infection with PEDV LJB/03 P23. PEDV LJB/03 was propagated in the Vero, Vero/TMPRSS2 and Vero/MSPL cells 3 serial passages, and the S genes, before and after infection with PEDV LJB/03, were amplified, sequenced and blasted. before: PEDV LJB/03 before infection; after: PEDV LJB/03 after infection.

**Fig. S2** Endogenous expression of TMPRSS2 and MSPL genes in the Vero cells.

the total RNA and complementary DNA (cDNA) of Vero cells were obtained and the presence of endogenously expressed TMPRSS2 and MSPL genes in Vero cells was examined. The experiment was repeated three times independently.
