# Supplementary figures and images for: Establishment of stable Vero cell lines expressing TMPRSS2 and MSPL: A useful tool for propagating porcine epidemic diarrhea virus in the absence of exogenous trypsin
Source: Virulence. 2020 May 29;11(1):669–85. doi: 10.1080/21505594.2020.1770491 (PMC7550007; doi:10.1080/21505594.2020.1770491)

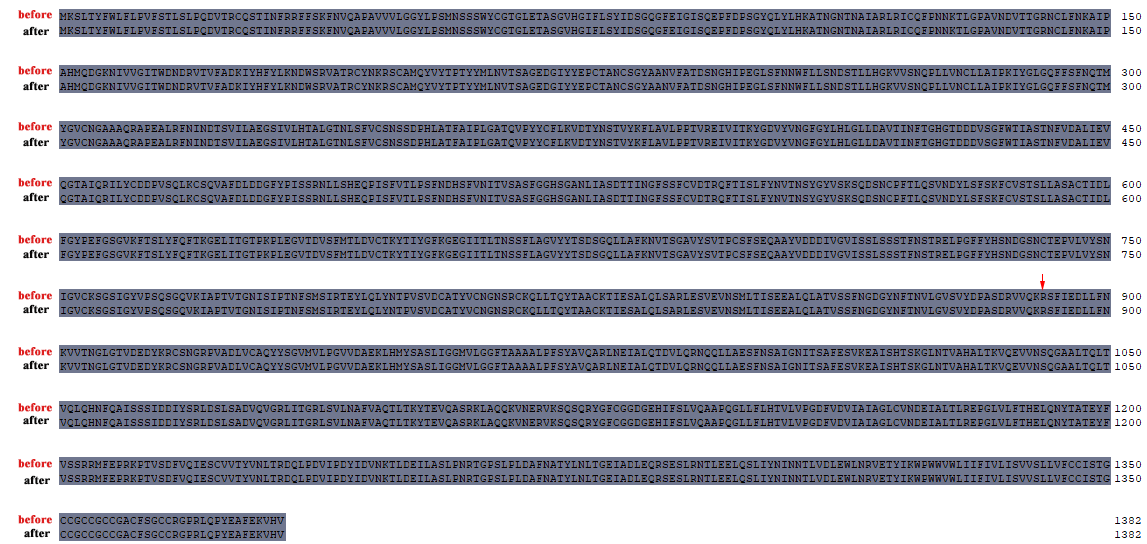

Supplement: Supplemental Material [file KVIR_A_1770491_SM8718.zip › Fig S1.tif]

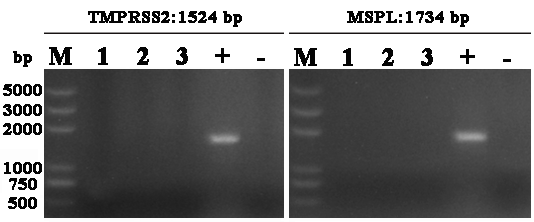

Supplement: Supplemental Material [file KVIR_A_1770491_SM8718.zip › Fig S2.tif]
